# Identifying key genes for diabetic kidney disease by bioinformatics analysis

**DOI:** 10.1186/s12882-023-03362-4

**Published:** 2023-10-18

**Authors:** Yushan Xu, Lan Li, Ping Tang, Jingrong Zhang, Ruxian Zhong, Jingmei Luo, Jie Lin, Lihua Zhang

**Affiliations:** 1 Department of Endocrinology, The First Affiliated Hospital of Kunming Medical University, Kunming, 650031 China; 2 Department of Diabetes, The First Affiliated Hospital of Kunming Medical University, Kunming, 650031 China; 3 Department of General Practice, The First Affiliated Hospital of Kunming Medical University, Kunming, 650031 China

**Keywords:** Diabetic Kidney Disease (DKD), Pathogenesis, mRNA microarray datasets, Differentially Expressed Genes (DEGs)

## Abstract

**Background:**

There are no reliable molecular targets for early diagnosis and effective treatment in the clinical management of diabetic kidney disease (DKD). To identify novel gene factors underlying the progression of DKD.

**Methods:**

The public transcriptomic datasets of the alloxan-induced DKD model and the streptozotocin-induced DKD model were retrieved to perform an integrative bioinformatic analysis of differentially expressed genes (DEGs) shared by two experimental animal models. The dominant biological processes and pathways associated with DEGs were identified through enrichment analysis. The expression changes of the key DEGs were validated in the classic *db/db* DKD mouse model.

**Results:**

The downregulated and upregulated genes in DKD models were uncovered from GSE139317 and GSE131221 microarray datasets. Enrichment analysis revealed that metabolic process, extracellular exosomes, and hydrolase activity are shared biological processes and molecular activity is altered in the DEGs. Importantly, *Hmgcs2, angptl4,* and* Slco1a1* displayed a consistent expression pattern across the two DKD models. In the classic *db/db* DKD mice, *Hmgcs2 and angptl4* were also found to be upregulated while *Slco1a1* was downregulated in comparison to the control animals.

**Conclusions:**

In summary, we identified the common biological processes and molecular activity being altered in two DKD experimental models, as well as the novel gene factors (*Hmgcs2, Angptl4,* and* Slco1a1)* which may be implicated in DKD. Future works are warranted to decipher the biological role of these genes in the pathogenesis of DKD.

**Supplementary Information:**

The online version contains supplementary material available at 10.1186/s12882-023-03362-4.

## Introduction

Diabetic kidney disease (DKD) is one of the most prevalent complications of diabetes and the main cause of end-stage renal disease (ESRD), accounting for ~ 50% of the total cases of ESRD in the United States and affecting approximately 30% of the global population [1–3]. Currently, there are no reliable molecular targets for early diagnosis and effective treatment in the clinical management of DKD [4, 5]. Therefore, identifying novel pathogenic factors closely associated with DKD development and progression not only facilitates the understanding of the molecular mechanisms of DKD, but also provides novel targets for therapeutic opportunities.

The etiology and pathogenesis of DKD is complex, which involves hierarchical physiological and pathological processes [3, 6, 7]. Different risk factors have been recognized as contributors to DKD, including lipid metabolic process [8], inflammation [9], oxidation–reduction process and ischemia [10] and oxidative stress [11]. Recent molecular and cellular researches explored new fields of pathophysiology of DKD, such as mitochondria dysfunction [12], podocyte autophagy [13], and genetic and epigenetic regulation [14]. Various types of animal models of DKD have been developed as valuable tools to investigate the pathogenesis of DKD and uncover important molecular events in the progression of DKD [15–17]. Nevertheless, different chemical inducers may incur a distinct physiological and pathological process, which may not be the direct driving factors or consequences of DKD. The identification of common gene factors helps define the biological changes shared by different models, and these genes are more likely to be disease-relevant and serve as a targetable factor for therapeutic interventions.

The rapid development of bioinformatic analysis has provided a platform to systematically survey the transcriptomic changes associated with disease conditions [18]. In this study, we retrieved and reanalyzed microarray datasets from the Gene Expression Omnibus (GEO), which are derived from an alloxan- or streptozotocin-induced DKD mouse models. We reported three previously uncharacterized genes, *Hmgcs2, Angptl4,* and *Slco1a1,* which showed consistent changes in the two animal models, and their expression changes were also verified in the classical *db/db* DKD mouse. Future functional characterization of these genes in DKD animal models will shed light on the unappreciated mechanisms of DKD progression.

## Materials and methods

### Gene expression omnibus dataset

Microarray data were downloaded from the GEO database (http://www.ncbi.nlm.nih.gov/geo). The GSE139317 dataset generated on the GPL21163 Agilent-074809 Sure-Print G3 Mouse GE v2 8 × 60 K Microarray platform comprises of 6 control and 9 DKD samples from alloxan-treated mice. The GSE131221 dataset derived from the GPL22740 Agilent-074036 Sure-Print G3 Rat GE v2 8 × 60 K Microarray G4858A platform contains 5 control and 7 DKD samples from streptozotocin-induced rats.

### Identification of differentially expressed genes (DEGs)

The Bioconductor R package was utilized for the differential gene expression analysis of the microarray data. Genes with a *p* value less than 0.05 and |log2-fold change (FC)| greater than 1.5 were considered the DEGs with statistical significance.

### Functional enrichment analysis

All identified DEGs in GSE139317 and GSE131221 were subjected to functional enrichment analysis by using Gene Ontology (GO) and Kyoto Encyclopedia of Genes and Genomes (KEGG) pathway database (https://www.kegg.jp/kegg/kegg1.html, KEGG is developed by Kanehisa Laboratories) [19]. For data annotation, DAVID bioinformatics resources (https://david.ncifcrf.gov/) was used for gene id conversion. Gene ontology enrichment analysis was performed using G:Profiler: a web server for functional enrichment analysis, with the threshold for significance at an adjusted *p* value < 0.05. In order to build a protein–protein interaction (PPI) network, we used the String database (https://string-db.org/) to retrieve the relationship among the candidates. The PPI network was constructed by Cytoscape software using a minimum Required Interaction score at 0.9 and with the parameter "hide unconnected nodes in the network.”

### Animal model of DKD

Male C57BL/6 mice (15 weeks; *n* = 5) and male *db/db* mice (15 weeks; *n* = 5) were purchased from Cavens Laboratory Animal Co., Ltd (Changzhou, China). The animals were fed with a standard rodent diet and water ad libitum. Diabetes mellitus in mice was defined as a blood glucose level > 16.7 mmol/L for 3 consecutive days, and the animals were euthanized after 16 weeks using a euthanizing chamber connected to a CO_2_ cylinder. HE staining was performed to verify the disrupted glomerular capillary structures, and PAS staining showed that the glomerular basement membrane was thickened and obvious glycogen deposition, which is the sign of DKD in the *db/db* mouse model [19].

### Quantitative real-time PCR

Total RNA extracted from the renal tissues using RNAiso Plus reagent (9109, TaKaRa, Kyoto, Japan). 1 μg of RNA sample was reverse-transcribed into complementary DNA using the first strand cDNA synthesis kit (RR047A; TaKaRa, Kyoto, Japan). Quantitative real-time PCR was conducted using SYBR green-based assay (11201ES03; Yeasen Biotech, Shanghai, China) on the CFX96 Real-Time system (Bio-Rad, Hercules, CA). β-actin gene was used as the internal reference for relative gene expression analysis based on the 2^−ΔΔCt^ method. The sequences of primers for real-time PCR are in the Table 1.

**Table 1:** Primers for qPCR in *db/db* mouse

Species	Gene name	Sequences 5’ → 3
Mouse	*Hmgcs2*	F: AGAGAGCGATGCAGGAAACTT
Mouse	*Hmgcs2*	R: AAGGATGCCCACATCTTTTGG
Mouse	*Angptl4*	F: CATCCTGGGACGAGATGAACT
Mouse	*Angptl4*	R: TGACAAGCGTTACCACAGGC
Mouse	*Slco1a1*	F: GATGAAGGTGTTTCTGATGTC
Mouse	*Slco1a1*	R: CCTTCCAAAATAACTCACG

### Statistics

Statistical analysis was performed using the GraphPad Prism software (USA). Data were presented as means ± standard deviation (SD). The significant differences were analyzed using the unpaired Student’s -t-test between the two groups. Data with *p* values of 0.05 or less were considered statistically significant.

## Results

### Project workflow

**Fig. 1:**
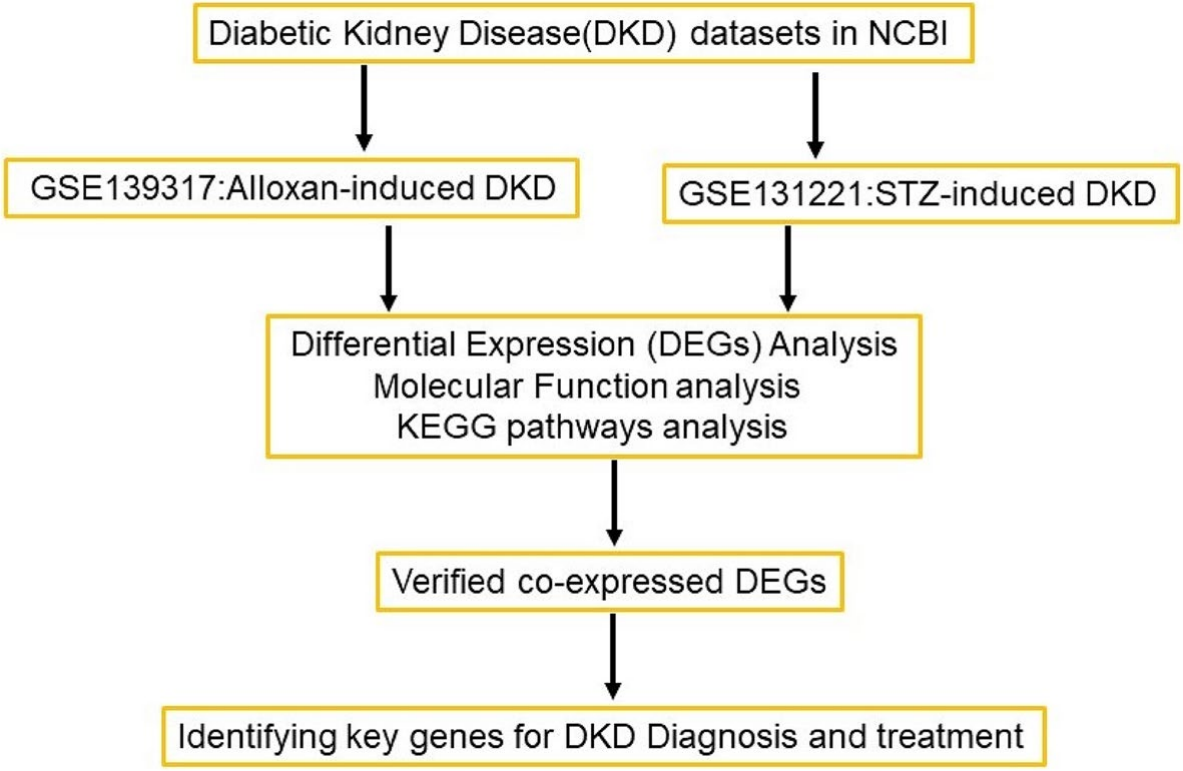
Study workflow. DKD, Diabetic kidney disease; STZ, streptozotocin; DEGs, differentially expressed genes; KEGG, Kyoto Encyclopedia of Genes and Genomes

In the current study, we formulated an integrative analysis plan (Fig. 1) to identify the potential pathogenic factors involved in DKD. A comparative analysis of microarray datasets derived from two different DKD models was performed to profile the DEGs, the associated biological processes, and KEGG pathway. The common DEGs of the two datasets were validated in the classical DKD model, which may be employed as candidates for diagnosis or treatment.

### Differential expression analysis of genes in alloxan-induced DKD model

**Fig. 2:**
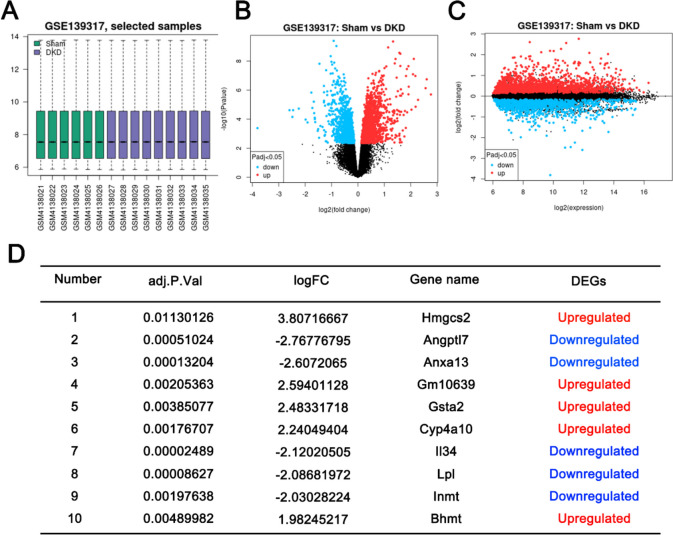
Differential expression analysis of GSE139317 dataset of alloxan-induced DKD model. **A** Boxplot presentation of gene expression distribution of 9 DKD and 6 sham samples. **B**, **C** Volcano map of DEGs. The DEGs were identified between the DKD group and the sham group with the criteria of |log2 FC| greater than 1 and the adjusted *P* value less than 0.05. **D** Top 10 DEGs identified between the DKD group and the sham group with the criteria of |log2 FC| greater than 1.5 and the adjusted *P* value less than 0.05

Alloxan-induced diabetic kidney malfunction was widely used to elucidate the pathophysiology of DKD. Gene expression profiles of 9 DKD and 6 sham samples were quality-checked and all data showed a median centered distribution (Fig. 2A), indicating they are statistically comparable. The volcano map in Fig. 2B, C displays the general distribution of gene expression changes, indicating an equivalent number of genes being upregulated or downregulated in DKD samples. In order to better screen the key genes with dramatic changes in DKD, we tightened the criteria of DEGs by using a |log2 FC| greater than 1.5 and an adjusted *P* value less than 0.05. As a result, 13 upregulated DEGs and 27 downregulated DEGs were identified between the DKD group and the sham group (shown in the Table 2), and the top 10 DEGs (*Hmgcs2, Angptl7, Anxa13, Gm10639, Gsta2, Cyp4a10, Il34, Lpl, Inmt, Bhmt*) were shown in Fig. 2D.

**Table 2:**
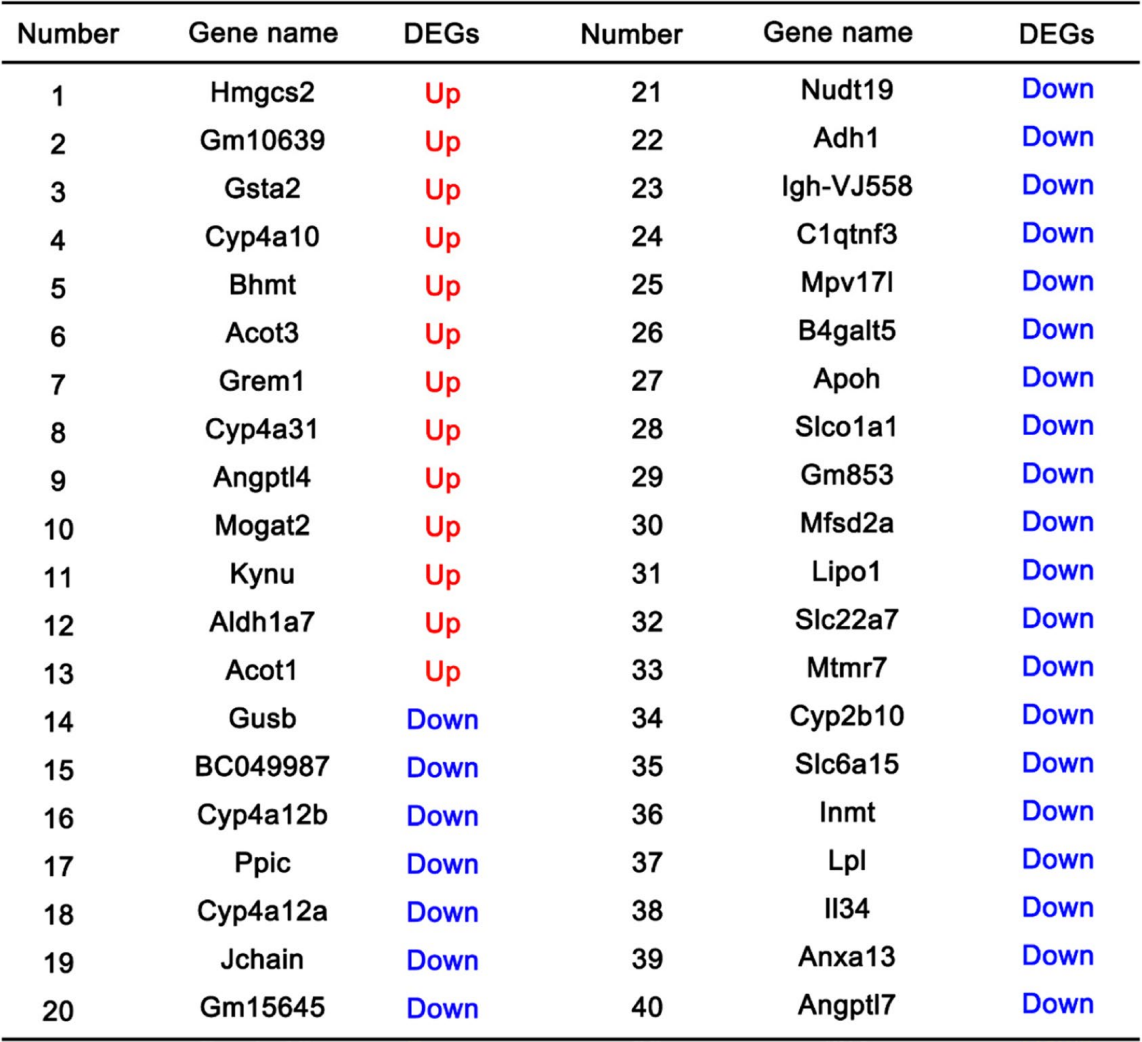
Upregulated and downregulated genes in alloxan-induced DKD

Blue indicated 27 downregulated genes and Red signified 13 upregulated genes, with the criteria of |log2 FC| greater than 1.5 and the adjusted *P* value less than 0.05

**Fig. 3:**
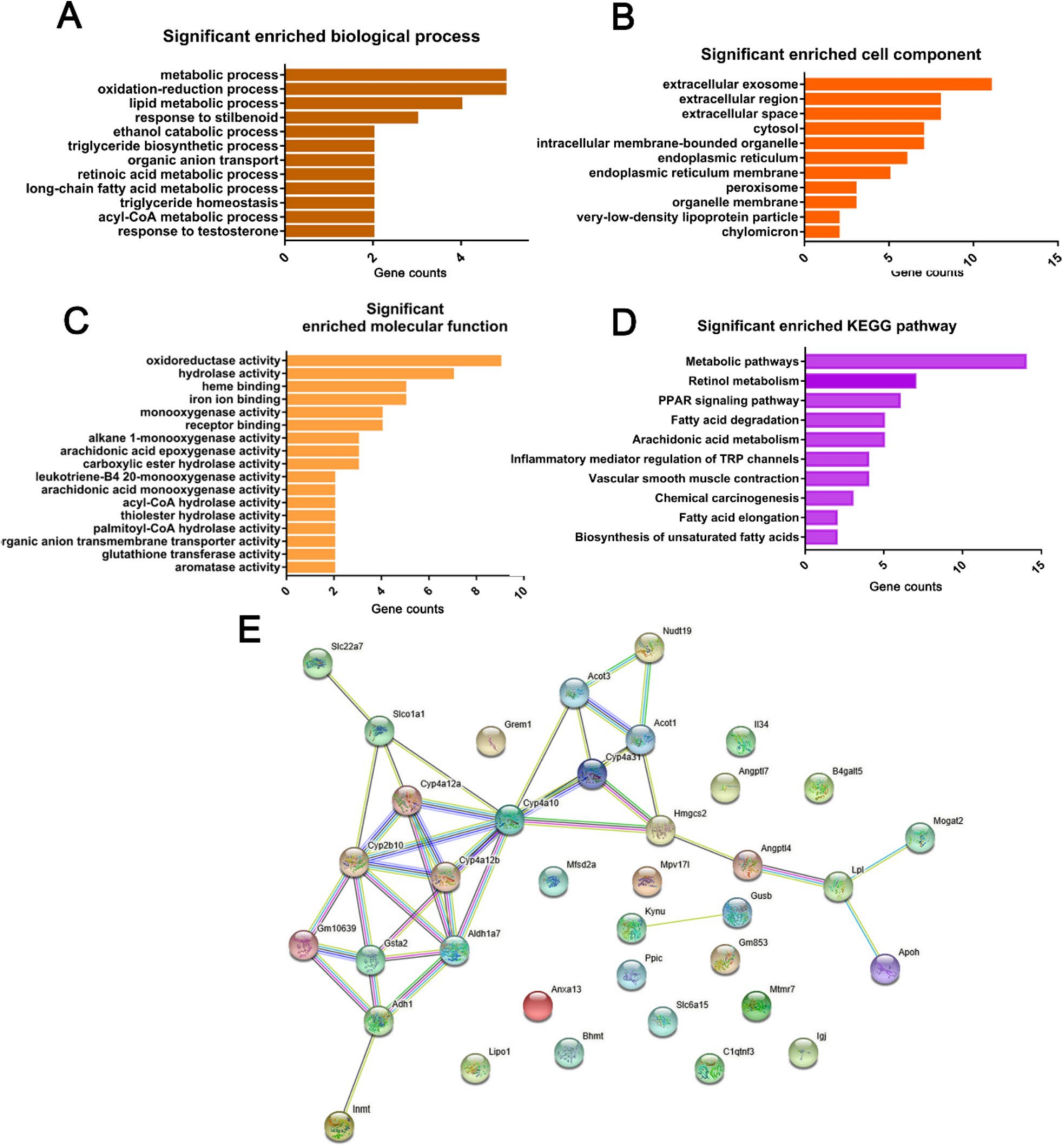
Functional enrichment analysis of the DEGs. **A-C** GO terms significantly enriched in the DGEs: BP, biological processes; CC, cellular component; MF, molecular function. **D** KEGG pathway enrichment analysis of DGEs. **E** Protein‐protein interaction network of DGEs. The above analyses were based on DEGs with the criteria of |log2 FC| greater than 1.5 and the adjusted *P* value less than 0.05

To gain more biological insights into the DEGs, functional enrichment analysis of the DEGs was performed to explore the Gene Ontology (GO) annotations and Kyoto Encyclopedia of Genes and Genomes (KEGG) pathways. The significantly enriched biological process mostly included metabolic process, oxidation–reduction process, lipid metabolic process, extracellular exosomes, extracellular region, hydrolase activity and heme binding (Fig. 3A-C). Metabolic pathways, retinol metabolism, PPAR signaling pathway and fatty acid degradation were the top KEGG pathways associated with DEGs (Fig. 3D). Furthermore, the protein–protein interaction (PPI) network analysis of the DGEs revealed a key module comprising of multiple members of the cytochrome P450 superfamily of enzymes (*Cyp2b10*, *Cyp4a10*, *Cyp4a12a*, *Cyp4a12b*), which are involved in the oxidation–reduction metabolism of fatty acids (Fig. 3E). Combining the above data, we conclude that the oxidation–reduction, metabolism, extracellular exosome, extracellular region, and hydrolase activity are the major biological process and cellular components altered in the alloxan-induced DKD model. The lipid-related metabolism suggests that DKD can be improved through diet and exercise, while the extracellular matrix suggests that fibrosis is also a point of concern in DKD.

### Profiling of DEGs in streptozotocin (STZ)-induced DKD model

**Fig. 4:**
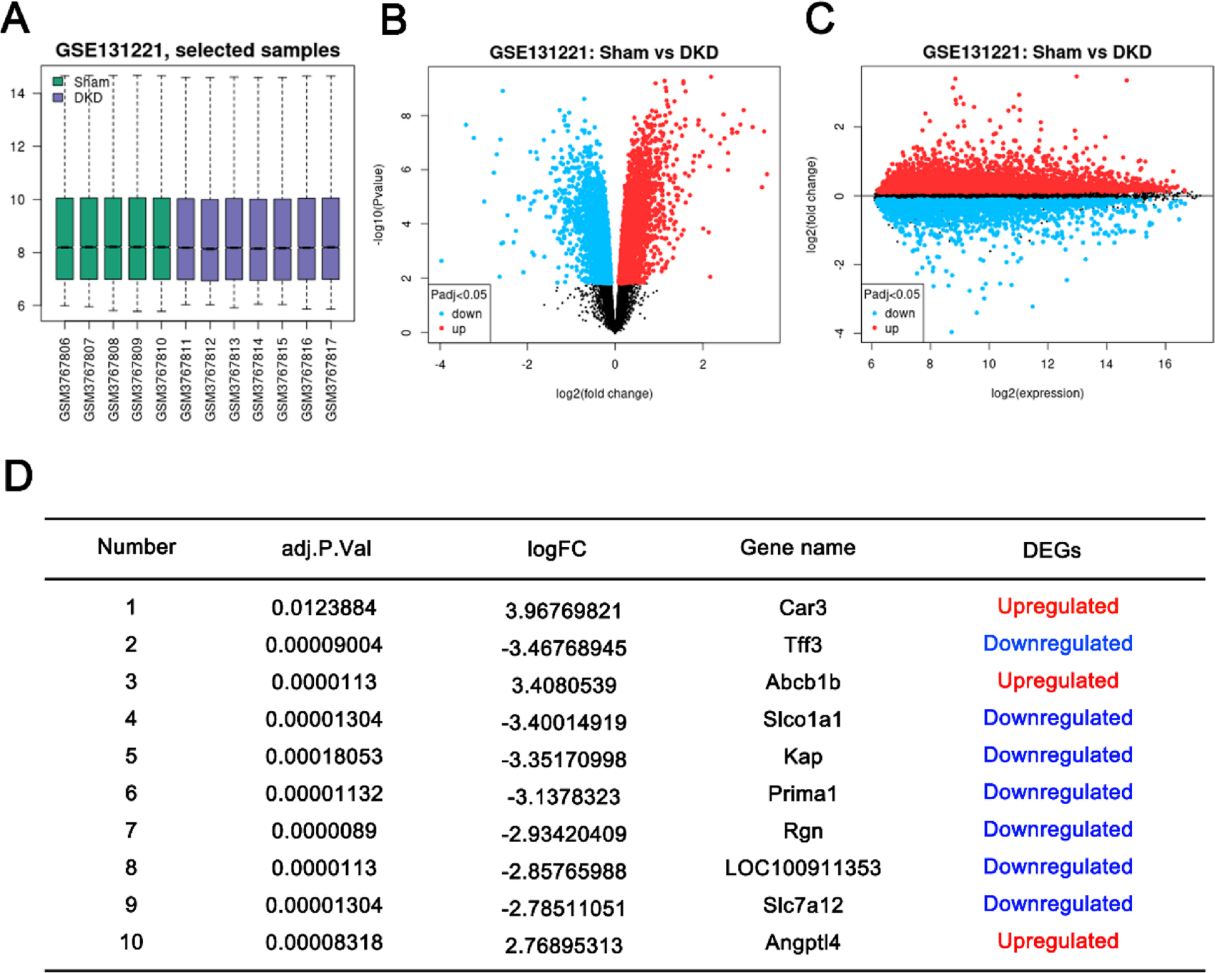
Differential expression analysis of GSE131221 dataset of streptozotocin (STZ)-induced DKD model. **A** Boxplot presentation of gene expression profiles of 7 DKD and 5 sham samples. **B**, **C** Volcano map of DEGs. The DEGs were identified between the DKD group and the sham group with the criteria of |log2 FC| greater than 1 and adjusted P value less than 0.05. **D** Top 10 DEG lists identified between the DKD and the sham group with the criteria of |log2 FC| greater than 1.5 and the adjusted *P* value less than 0.05

**Fig. 5:**
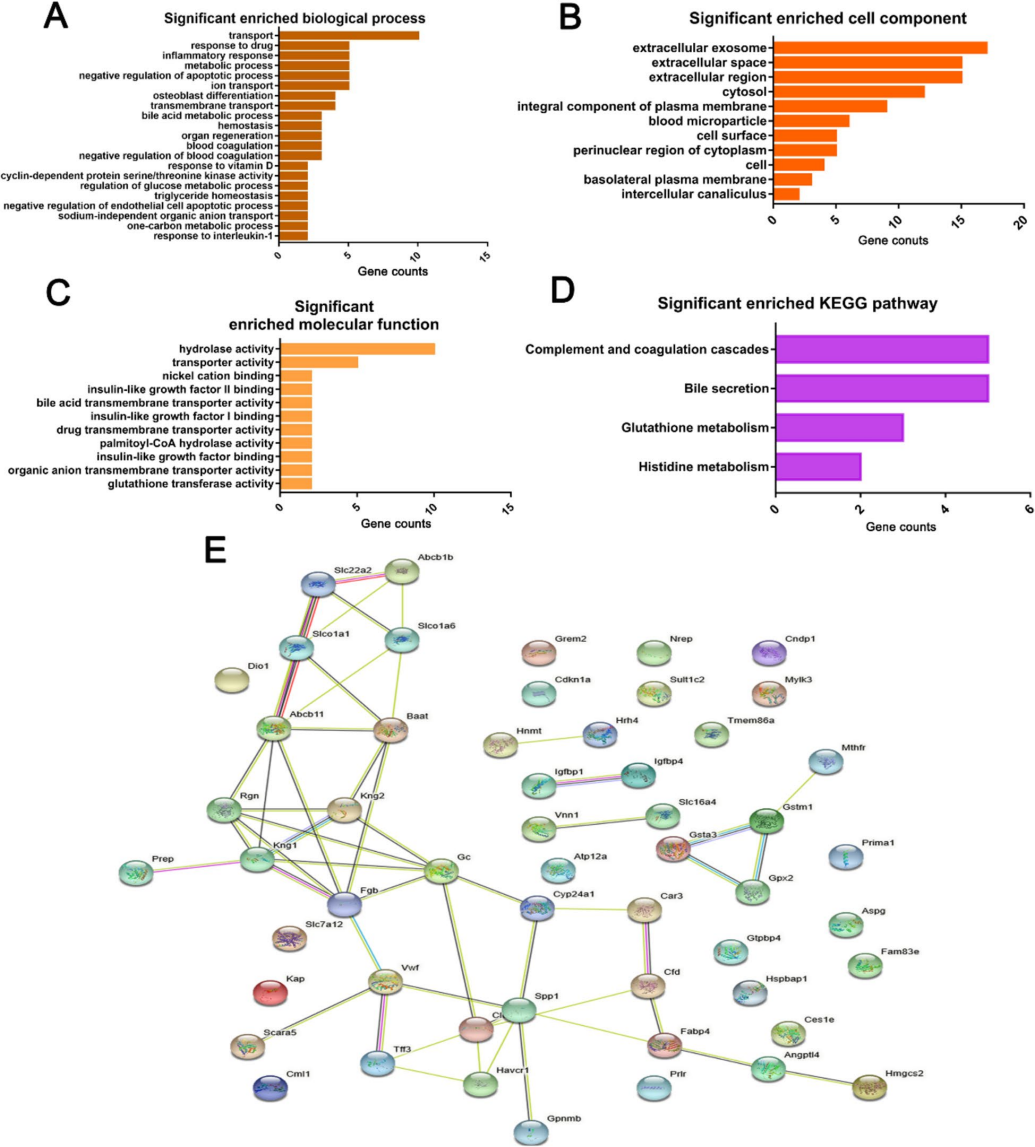
Enrichment analysis. **A-C** GO terms significantly enriched in the DGEs: BP, biological processes; CC, cellular component; MF, molecular function. **D** KEGG pathway enrichment analysis related to DGEs. **E** Protein‐protein interaction networks related to DGEs

Next, we profiled the DEGs in streptozotocin (STZ)-induced DKD model. The dataset comprises of 7 DKD and 5 sham samples (Fig. 4A), in which **t**he DEGs were initially identified with the criteria of |log2 FC| greater than 1 and the adjusted *P* value less than 0.05 (Fig. 4B, C). Likewise, the criteria of |log2 FC| greater than 1.5 and the adjusted *P* value less than 0.05 were further used to identify the DEGs with dramatic changes. 27 upregulated genes and 34 downregulated genes between the DKD and the sham groups were identified (shown in the Table 3), and the top 10 DEGs (*Car3, Tff3, Abcb1b, Slco1a1, Kap, Prima1, Rgn, LOC100911353, Slc7a12, Angptl4*) were shown in Fig. 4D. In this regard, the GO and KEGG enrichment analysis showed a broad spectrum of biological processes and pathways were associated with the DEGs, including the response to drugs, inflammatory response, metabolic process, negative regulation of apoptotic process, ion transport, extracellular exosomes, extracellular space, hydrolase activity, complement and coagulation cascade, bile secretion, glutathione metabolism and histidine metabolism (Fig. 5A-D). Consistent with these data, PPI network indicated a wide range of DEGs involved in different cellular processes were implicated in STZ-induced DKD (Fig. 5E). Together, these data indicate that except for the extracellular exosome, extracellular space, and hydrolase activity, there are other biological processes such as complement and coagulation cascade, bile secretion, glutathione metabolism and histidine metabolism that are affected in the pathophysiological process of STZ-induced DKD model.

**Table 3:**
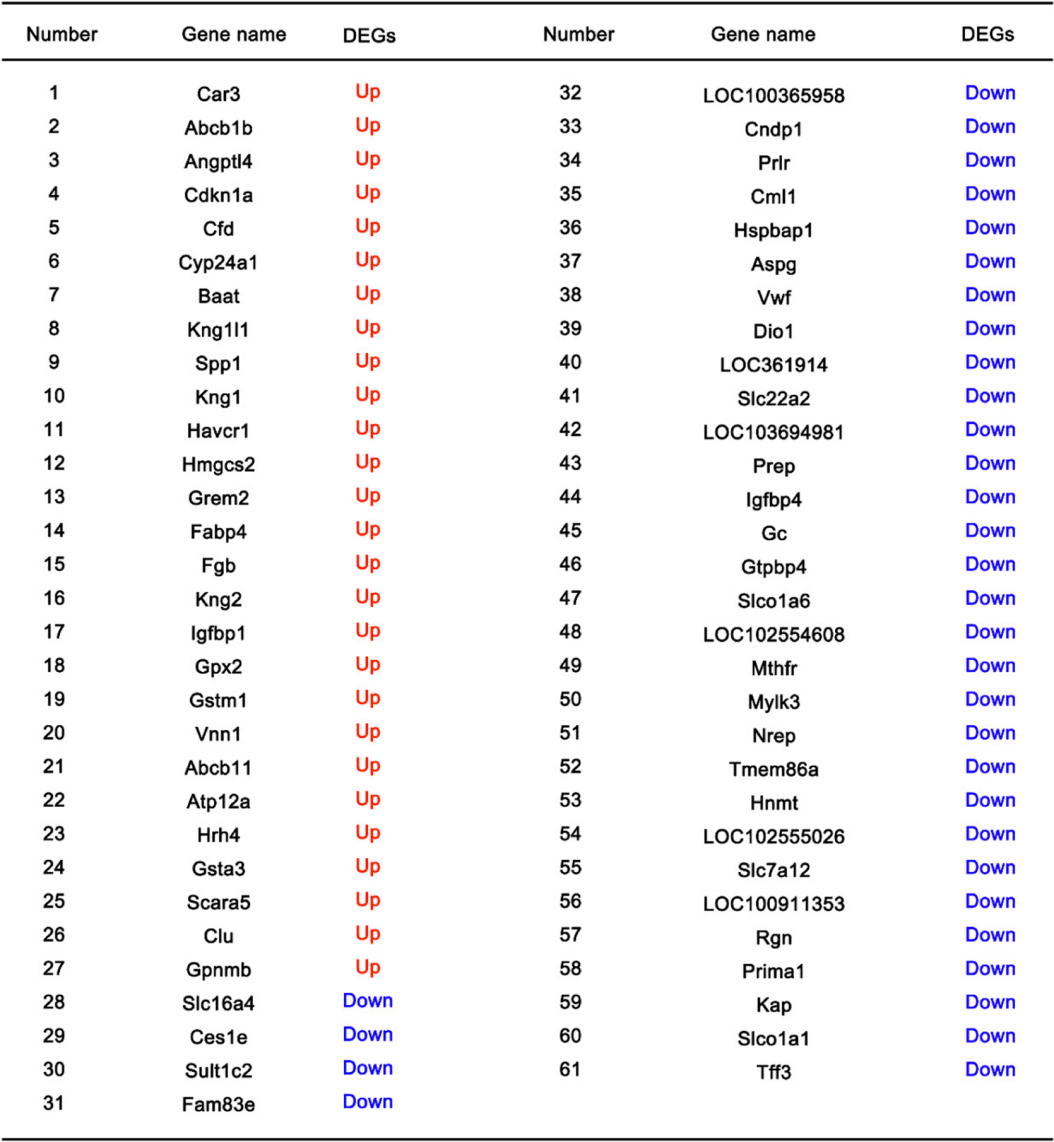
Upregulated and downregulated DEGs in STZ-induced DKD

Blue indicated 34 downregulated genes and Red indicated 27 upregulated genes, with the criteria of |log2 FC| greater than 1.5 and the adjusted *P* value less than 0.05

### Identifying the common DEGs in two DKD models

**Fig. 6:**
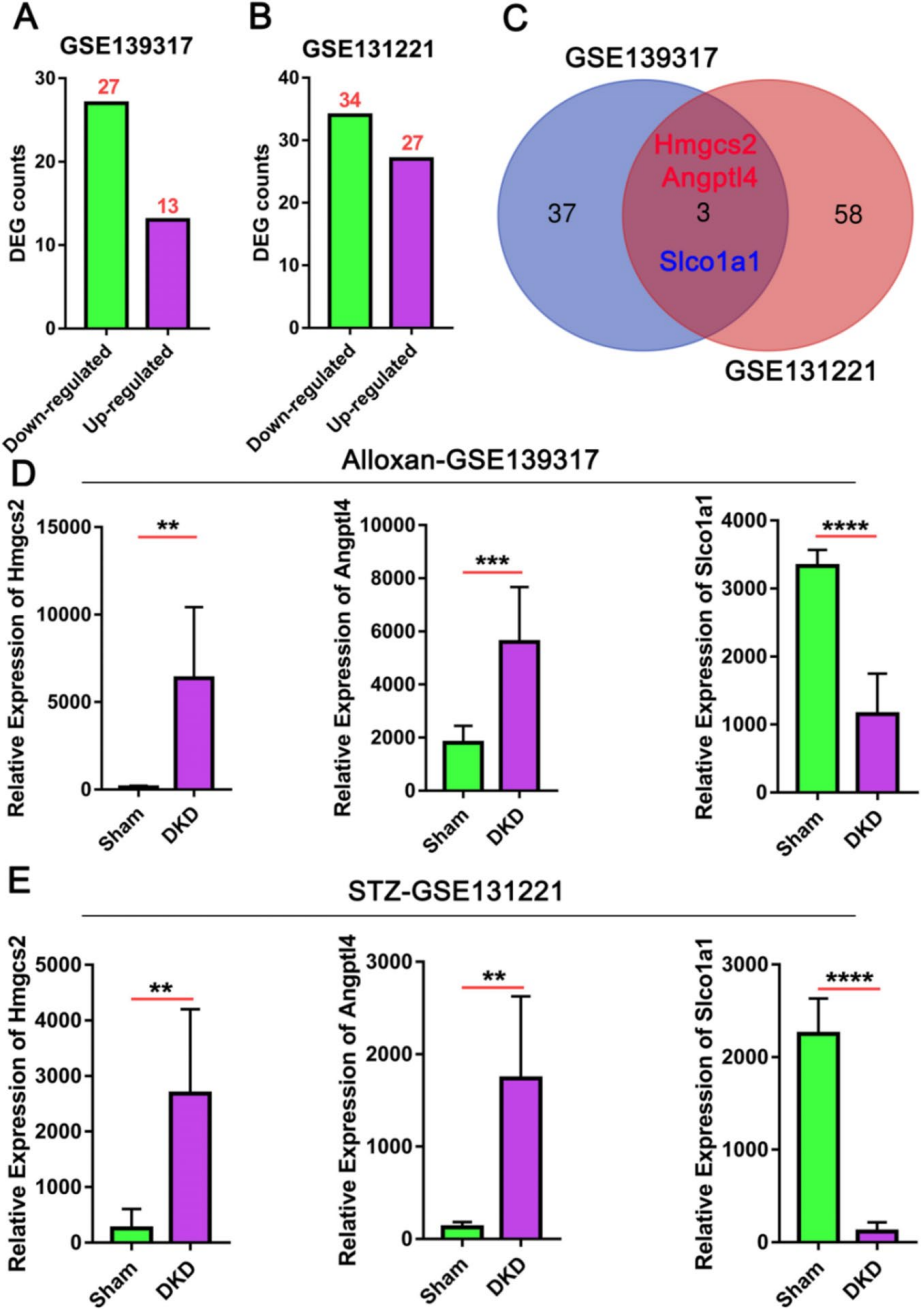
The identification of common DEGs affected in the two DKD models. **A**, **B** The number of DEGs was significantly upregulated or downregulated in GSE139317 or GSE131221, with the criteria of |log2 FC| greater than 1.5 and the adjusted *P* value less than 0.05. **C** The Venn diagram shows the common DEGs of GSE139317 and GSE131221 datasets. **D**, **E** The relative expression of *Hmgcs2, Angptl4,* and* Slco1a1* in alloxan-induced model (GSE139317) and STZ-induced DKD model (GSE131221). *P < 0.05, **P < 0.01, ***P < 0.001

The above analyses demonstrated that although there are common biological processes affected in both models of DKD, there are specific processes exclusively enriched in each model, some changes incurred may be unique to the chemical inducer in alloxan and STZ-induced DKD. These changes are unlikely to be the true signals signifying the pathogenic process of DKD. To find the gene factors shared by the two models, we analyzed the intersection of the downregulated and upregulated genes with the criteria of |log2 FC| greater than 1.5 and the adjusted *P* value less than 0.05. (Fig. 6A, B). Three genes, *Hmgcs2, Angptl4,* and *Slco1a1* displayed a consistent change in both alloxan and STZ-induced DKD models (Fig. 6C). As shown in Fig. 6D, E, *Hmgcs2* and *Angptl4* showed dramatic upregulation while *Slco1a1* expression was suppressed in both DKD models. Relevant to our analyses, the activity of *Hmgcs2* in the kidneys and 24-h urinary excretion of the ketone body β-hydroxybutyrate (β-HB) were reported to be increased in db/db mice [20]. The upregulation of *Angptl4* was found in the development of diabetic nephropathy [21], indicating a potential role for *Angptl4* in DN for the detection of a diabetic kidney disease [22] and as a therapeutic target [23]. Consistently, a previous study reported that TNF-α and TGF-β1 downregulated *Slco1a*1 in primary hepatocytes, suggesting a proinflammatory role [24] and hypermethylation of *Slco1a1* locus in diabetic mice was observed [25]. Taken together, our analyses suggest the potential contribution of *Hmgcs2, Angptl4,* and *Slco1a1* to the pathogenesis of DKD, and the functional characterization of these genes is conducive to the understanding of the underlying mechanisms of DKD.

### Validation of the expression change of Hmgcs2, Angptl4 and Slco1a1 in db/ db DKD mice

**Fig. 7:**
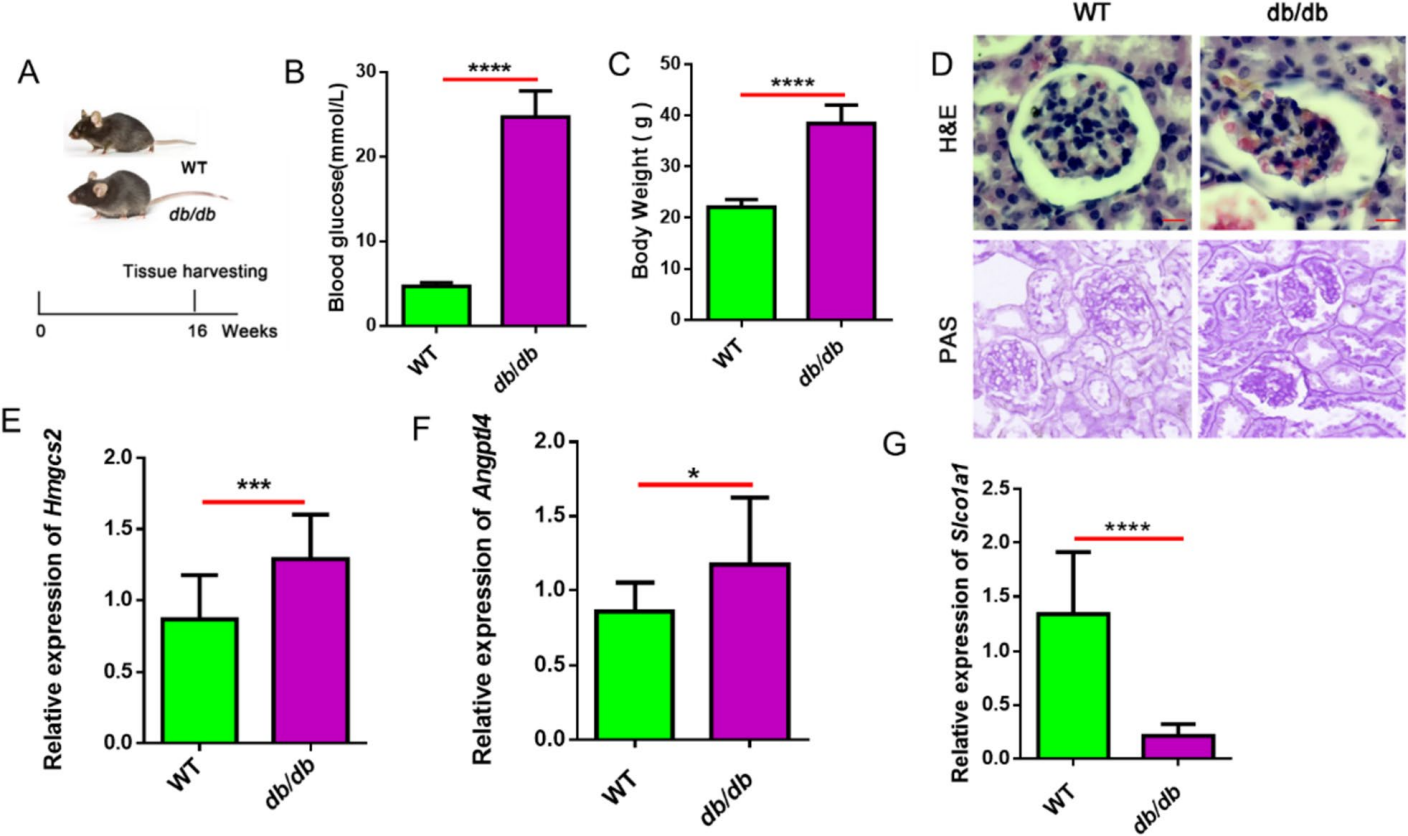
The expression of *Hmgcs2, Angptl4,* and* Slco1a1* in *db/db* mice. **A** Schematic illustration of the experimental design. **B** The blood glucose levels in *db/db* mice and WT mice were measured at week 16 (*n* = 5 per group). **C** The body weights in *db/db* mouse, *and b/db* mouse, and WT mice were measured at week 16 (*n* = 5 per group). **D-F** The relative expressions of *Hmgcs2, Angptl4,* and* Slco1a1* in *db/db* mice and *b/db* mice and WT mice were measured at week 16. *P < 0.05, **P < 0.01, ***P < 0.001

To further confirm the changes of these genes, we used the *db/db* mice as the well-established animal model of DKD study [26], to detect the expression pattern of *Hmgcs2, Angptl4* and *Slco1a1* (Fig. 7A). To confirm the onset of diabetic symptoms, we measured the blood glucose level (Fig. 7B) and body weight (Fig. 7C) of the wildtype controls (WT) and *db/db* mice at the end of week 16, which showed an elevated blood glucose level and gain in body weight. H&E and PAS staining of renal tissue sections showed the disrupted glomerular capillary structures, verifying the onset of DKD in the db/db mouse model (Fig. 7D). Compared with the WT mice, the expression of *Hmgcs2* (Fig. 7E) and* Angptl4* (Fig. 7F) were significantly increased in the renal tissues of *db/db* mice, while the expression of *Slco1a1* (Fig. 7G) was suppressed in *db/db* mice. Taken together, these data further support the uncharacterized roles of *Hmgcs2, Angptl4,* and *Slco1a1* in the pathogenesis of DKD.

## Discussion

Despite intense efforts to understand the signaling pathways and molecular players driving DKD, the pathogenesis of DKD remains as an intricate process. Because of the fact that a single DKD model may not comprehensively reflect the true signals underlying DKD progression, the integrative analysis of different models could provide an unbiased view of the biological processes and key genes contributing to the progression of DKD. In the current study, we conducted in-depth analysis of the transcriptome data of two widely used DKD models (alloxan-induced model and streptozotocin-induced model). We revealed the common and unique DEGs and biological processes altered in these two DKD models. Most importantly, three genes, *Slco1a1, Angptl4,* and *Hmgcs2* displayed a consistent change across the two models, which was further verified in *db/db* mice, implying an important role of *Slco1a1, Angptl4* and *Hmgcs2* as candidate targets for the diagnosis and intervention of DKD.

The pathogenesis of DKD is complex, which is driven by both genetic and environmental factors [27, 28]. Several genes have been proposed to influence the initiation and development of DKD. The C-X-C motif chemokine ligand 1 (CXCL1) was indicated to serve as a proinflammatory mediatorin the DKD [29]. In addition, von willebrand factor (VWF) involved in intrarenal thrombosis was suggested to mediate the deterioration of renal function [30]. Importantly, Spleen tyrosine kinase (SYK) is a protein kinase mediating the secretion of high glucose-induced IL-1β [31] and TGF-β [32]. Metabolic changes associated with obesity, diabetes, aging, and nutritional status also play critical roles in DKD development [33]. Furthermore, studies reported that effective control of smoking, blood pressure, blood glucose, and lipid intake could significantly improve the DKD [34]. However, given the complexity of DKD and the limited strategies of treatment in human patients [35, 36], it is imperative to uncover novel molecular players and develop targeted therapies for DKD management.

Alloxan and streptozotocin (STZ) are the most widely used chemicals to induce DKD in animal models [15, 37]. However, alloxan and streptozotocin can each trigger gene expression changes which are irrelevant to the DKD, thus the biased conclusion may not reflect the true signals of DKD in a single animal model. For example, our analysis demonstrated that the GO annotations and KEGG pathway of DEGs in alloxan-induced DKD are mainly involved in metabolic process, oxidation–reduction process, lipid metabolic process, response to stilbenoid, ethanol catabolic process, triglyceride biosynthetic process, metabolic pathways, retinol metabolism, PPAR signaling pathway and fatty acid degradation. While, in the STZ-induced DKD, the mainly significant enriched biological process includes transport, response to drug, inflammatory response, metabolic process, extracellular exosomes, extracellular space, hydrolase activity, negative regulation of apoptotic process, ion transport, complement and coagulation cascades, bile secretion, glutathione metabolism, histidine metabolism. Therefore, the common processes affected in both models are the metabolic processes, extracellular exosome, extracellular space and hydrolase activity in both models.

Through our PPI network analysis, several members of the network were previously linked to DKD progression, such as *Slc22a1* [38], *Cyp24a1* [39], *Gstm1* [40] and *Vwf* [41]. Among them, *Hmgcs2, Angptl4,* and* Slco1a1* were the genes of interest that are commonly affected in alloxan and STZ-induced DKD. The activity of *Hmgcs2* in the kidneys and 24-h urinary excretion of the ketone body β-hydroxybutyrate (β-HB) were found to be increased in *db/db* mice [19, 20]. Upregulation of *Angptl4* in diabetic nephropathy was proposed to contribute to the development of diabetic nephropathy [21], indicating a potential role of *Angptl4* for the detection of a diabetic kidney disease [22] and as a potential therapeutic target [23]. Furthermore, a previous study reported that TNF-α and TGF-β1 downregulate *Slco1a*1 in primary hepatocytes to modulate hepatic fibrosis and inflammation, suggesting an proinflammatory role of *Slco1a*1 [24], and the hypermethylation of *Slco1a1* locus in diabetic mice was observed [25]. Given the fact that these genes are commonly altered in two different DKD models and their potential functional engagement in DKD progression, future works are warranted to functionally characterize their roles in DKD to provide insights into the molecular mechanisms of DKD and help formulate novel intervention strategies.

In summary, the current study identified *Hmgcs2, Angptl4,* and* Slco1a1* as critical genes in the pathogenesis of two DKD animal models, which are of significance in the understanding of the underlying mechanisms of DKD and may serve as promising targets for DKD clinical management. However, further verification of these genes in clinical samples and functional characterization are needed to confirm their implications in DKD.

### Supplementary Information

**Additional file 1:** **Supporting Table S1.** Primers for qPCR in *db/db* mouse.

## Data Availability

The datasets used and analyzed during the current study available from the corresponding author on reasonable request.
