# Supplementary material for: Identifying key genes for diabetic kidney disease by bioinformatics analysis
Source: BMC Nephrol. 2023 Oct 18;24:305. doi: 10.1186/s12882-023-03362-4 (PMC10585855; doi:10.1186/s12882-023-03362-4)
Supplement: Supplementary file 1 — Additional file 1: Supporting Table S1. Primers for qPCR in db/db mouse. [file 12882_2023_3362_MOESM1_ESM.docx]

**Supporting Table**

**Supporting** **Table S1. Primers for qPCR in *db/db* mouse**

| Species | Gene name | Sequences 5’→3 |
| --- | --- | --- |
| Mouse | *Hmgcs2* | F: AGAGAGCGATGCAGGAAACTT |
| Mouse | *Hmgcs2* | R: AAGGATGCCCACATCTTTTGG |
| Mouse | *Angptl4* | F: CATCCTGGGACGAGATGAACT |
| Mouse | *Angptl4* | R: TGACAAGCGTTACCACAGGC |
| Mouse | *Slco1a1* | F: GATGAAGGTGTTTCTGATGTC |
| Mouse | *Slco1a1* | R: CCTTCCAAAATAACTCACG |
